# Blood metabolomics improves prediction of central nervous system damage in multiple sclerosis

**DOI:** 10.1007/s11306-025-02315-2

**Published:** 2025-08-12

**Authors:** Jessica Rebeaud, Nicholas Edward Phillips, Guillaume Thévoz, Solenne Vigne, Sedreh Nassirnia, Aude Gauthier-Jaques, Pansy Lim-Dubois-Ferriere, Satchidananda Panda, Marie Théaudin, Renaud Du Pasquier, Gilbert Greub, Claire Bertelli, Jens Kuhle, Tinh-Hai Collet, Caroline Pot

**Affiliations:** 1 Laboratories of Neuroimmunology, Department of Clinical Neurosciences, Center for Research in Neuroscience and Service of Neurology, Lausanne University Hospital and University of Lausanne, Lausanne, Switzerland; 2 Service of Endocrinology and Diabetology, Geneva University Hospitals, Geneva, Switzerland; 3 Institute of Microbiology, Lausanne University Hospital and University of Lausanne, Lausanne, Switzerland; 4 Salk Institute for Biological Studies, La Jolla, CA 92037 USA; 5 Departments of Biomedicine and Clinical Research, Multiple Sclerosis Centre and Research Center for Clinical Neuroimmunology and Neuroscience (RC2NB), University Hospital and University of Basel, Basel, Switzerland; 6 Department of Neurology, University Hospital and University of Basel, Basel, Switzerland; 7 Faculty of Medicine, Diabetes Centre, University of Geneva, Geneva, Switzerland

**Keywords:** Multiple sclerosis, Metabolomics, Gut-microbiota, Biomarkers

## Abstract

**Introduction:**

Multiple sclerosis (MS) is an autoimmune disorder with an unpredictable outcome at the time of diagnosis. The measurement of serum neurofilament light chain (sNfL) and glial fibrillary acidic protein (sGFAP) has introduced new biomarkers for assessing MS disease activity and progression. However, there is a need for additional diagnostic and prognostic tools. In this study, we investigated the predictive abilities of metabolomics, gut microbiota, as well as clinical and lifestyle factors for MS outcome parameters.

**Objectives:**

The aim of this study was to assess the predictive capacity of plasma metabolites, gut microbiota, and clinical/lifestyle factors on MS outcome measures including MS-related fatigue, MS disability, and sNfL and sGFAP concentrations.

**Methods:**

A prospective cohort study was conducted with 54 individuals with MS. Anthropometric, biological, and lifestyle parameters were collected. The least absolute shrinkage and selection operator (LASSO) algorithm with ten-fold cross-validation was used to identify predictors of MS disease outcome parameters based on plasma metabolomics, microbiota sequencing, and clinical and lifestyle measurements obtained from questionnaires and anthropometric measurements.

**Results:**

Circulating metabolites were found to be superior predictors for sNfL and sGFAP concentrations, while clinical and lifestyle data were associated with EDSS scores. Both plasma metabolites and clinical data significantly predicted MS-related fatigue. Combining multiple multi-omics data did not consistently improve predictive performance.

**Conclusions:**

This study highlights the value of plasma metabolites as predictors of sNfL, sGFAP, and fatigue in MS. Our findings suggest that prioritizing metabolomics over other methods can lead to more accurate predictions of MS disease outcomes.

**Graphical abstract:**

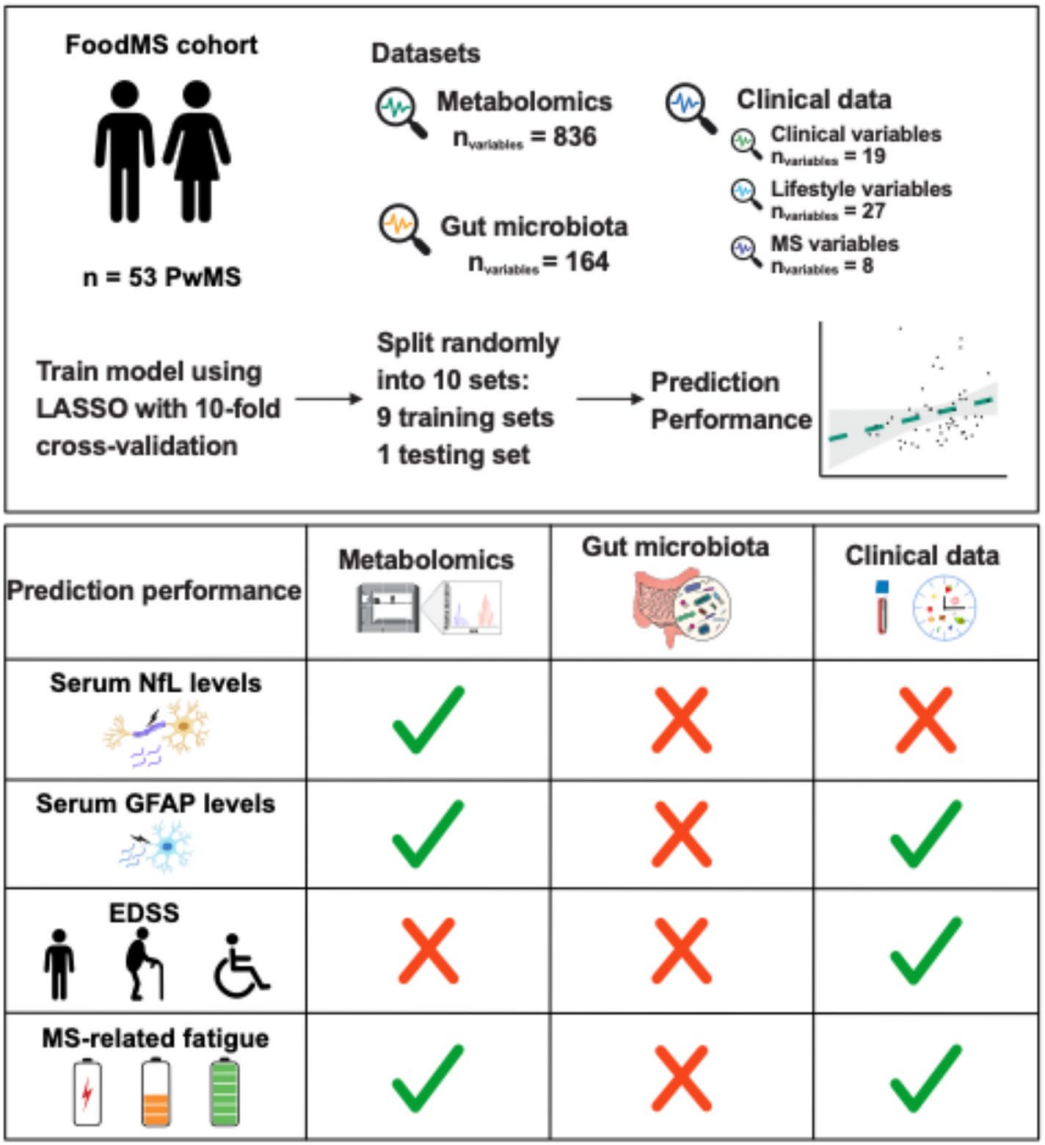

**Supplementary Information:**

The online version contains supplementary material available at 10.1007/s11306-025-02315-2.

## Introduction

Multiple sclerosis (MS) is a prevalent and debilitating autoimmune neurological disease that affects young individuals. It is mediated by the infiltration of self-reactive myelin-specific T cells in the central nervous system (CNS), leading to chronic demyelination and axonal damage.

Genetic and environmental factors contribute to the development of MS. Environmental risk factors include smoking, viral infections, low vitamin D intake, obesity during adolescence, and changes in the gut microbiota (Olsson et al., 2017). The gut-brain axis and the gut microbiota influence the course of MS (Jangi et al., 2016). Persons with MS (PwMS) display gut microbiota dysbiosis compared to controls (Miyake et al., 2015). Various mechanisms including the vagus nerve, immune cell trafficking, and microbiota-derived metabolites contribute to the gut-brain axis. Since microbiota-derived metabolites can be measured in the blood, plasma metabolomics represents a promising avenue for studying the gut-brain axis. A metabolomic signature was proposed to classify PwMS with an Expanded Disability Status Scale (EDSS) > 3 and to differentiate PwMS from controls (Villoslada et al., 2017). However, the predictive potential of metabolomics for disease evolution is not yet known. Serum neurofilament light chain (sNfL) and glial fibrillary acidic protein (sGFAP) are proposed as potential biomarkers to monitor the course of MS (Kuhle et al., 2016; Meier et al., 2023) and of its murine model. Their levels are increased following experimental autoimmune encephalomyelitis (EAE) induction (Zahoor et al., 2025). sNfL and sGFAP are markers of neuronal and astrocytic degeneration, respectively. sNfL correlates with white matter lesion volume (Kuhle et al., 2016), clinical and MRI disease activity measurements, and has prognostic value (Kuhle et al., 2019). sGFAP may be complementary to sNfL and could be a marker of MS disease progression (Meier et al., 2023).

It is unknown which other factors could predict axonal damage and recovery biomarkers in MS. Our study assessed the ability of clinical data, gut microbiota, and blood metabolomics to predict MS disease severity parameters, focusing on EDSS, MS-related fatigue scores, and blood biomarkers sNfL and sGFAP.

## Methods

### Population and eligibility criteria

In this observational prospective study, 54 PwMS were recruited from the CoolinBrain cohort (CER-VD 2018–01622) after their MS diagnosis was confirmed based on the McDonald 2017 criteria (Thompson et al., 2018). Each participant signed a written informed consent form approved by the local Ethics Committee (CER-VD 2018–01862) before being included in the study.

To be eligible, participants had to be adults older than 18 years, with a body mass index (BMI) ≥ 18 kg/m^2^, stable weight (± 2 kg) over the previous 3 months, and users of a smartphone compatible with the research-dedicated application MyCircadianClock (Gill & Panda, 2015). Pregnant and breastfeeding women, individuals on a diet, with eating or sleep disorders, who had undergone prior bariatric surgery, shift workers, or traveling to another time zone during the study were excluded.

### Clinical data collection

We collected clinical data as previously described (Thévoz et al., 2024). The datasets were divided into clinical, lifestyle, and MS variables, which are summarized in Table 1.

**Table 1:** Clinical datasets listing every variable in three categories

Clinical data		
Clinical and metabolic health variables	Lifestyle and dietary variables	MS related disability variables
**Anthropometric variables**	**Sleep and circadian rhythm variables**	**Clinical disease measures**
Body mass index	Sleep duration	EDSS
Waist-to-hip ratio	Abs. (Sleep duration—7.5 h)	MS duration
Waist-to-height ratio	PSQI	DMT class
**Vital signs**	Eating midpoint^a^	**Biomarkers**
Age	Abs. (Eating midpoint—14 h)^a^	sNfL concentration
Sex	Eating start^a^	sGFAP concentration
Systolic blood pressure	Eating stop^a^	**Patient-reported outcomes**
Diastolic blood pressure	Eating duration^a^	EMIF-SEP cognitive dimension
**Basic biochemistry laboratory measures**	**Dietary intake and food quality**	EMIF-SEP physical dimension
Leucocytes	Proportion of NOVA1 entries (%)^a^	EMIF-SEP social dimension
Hemoglobin	Proportion of NOVA4 entries (%)^a^	EMIF-SEP psychological dimension
Fasting plasma glucose	Energy intake^b^	EMIF-SEP score total
Total cholesterol	Protein intake^b^	
HDL cholesterol	Carbohydrate intake^b^	
LDL cholesterol	Fat intake^b^	
Triglycerides	Short fatty acid intake^b^	
ASAT	Mono-insaturated FA intake^b^	
ALAT	Poly-insaturated FA intake^b^	
Alkaline phosphatase	Red meat per month^b^	
Albumin	Vegetable score^b^	
Ferritin	Fruit score^b^	
eGFR	Nut-soy score^b^	
HbA1c	Cereal score^b^	
	Ratio of polyunsaturated to saturated fat^b^	
	Vitamin score^b^	
	Alcohol intake^b^	
	Alternative healthy eating index^b^	
	**Physical activity**	
	IPAQ score	

*Abs* Absolute, *HDL* High-density lipoprotein, *LDL* Low-density lipoprotein, *ASAT* Aspartate aminotransferase, *ALAT* Alanine aminotransferase, *eGFR* Estimated glomerular filtration rate, *HbA1c* Glycosylated hemoglobin, *PSQI* Pittsburgh sleep quality index, *IPAQ* International physical activity questionnaire, *FA* Fatty acid, *EDSS* Expanded Disability Status Scale, *MS* Multiple sclerosis, *DMT* Disease-modifying therapies, *sNfL* Serum neurofilament light chain, *sGFAP* Serum glial fibrillary protein, *EMIF-SEP* Échelle de mesure de l’impact de la fatigue dans la sclérose en plaques; Scale for measuring the impact of fatigue in multiple sclerosis

^a^Calculated based on food and drink timestamps collected from the MyCircadianClock app

^b^Calculated based on food frequency questionnaires

#### Clinical and metabolic health variables

At baseline, we collected socio-demographic and biological parameters. BMI was calculated by dividing weight in kilograms by height in meters squared, using calibrated scales and a stadiometer. Waist circumference (WC) was measured between the 12th ribs and the iliac crest, while hip circumference was measured at the level of the greater trochanters. Waist-to-hip ratio (WHR) was determined by dividing WC by hip circumference, and waist-to-height ratio (WtHR) by dividing WC by height. Fasting blood samples were analyzed for cholesterol, triglycerides, liver and renal function, plasma glucose, glycated hemoglobin (HbA1c), and full blood count.

#### Lifestyle and dietary variables

Sleep quality was evaluated using the Pittsburgh Sleep Quality Index (PSQI) (Buysse et al., 1989) and chronotype with the Munich Chronotype Questionnaire (Roenneberg et al., 2003). Physical activity was assessed using the International Physical Activity Questionnaire (IPAQ) short form (Craig et al., 2003), which considers walking, moderate, and vigorous physical activity over the past 7 days estimating total activity in METs-min/week. Participants were asked to photograph all food and drink intake over 4 weeks using the MyCircadianClock smartphone app (Gill & Panda, 2015). Eating duration was defined as the time between the 2.5th and 97.5th percentiles of all timestamped ingestion events, calculated from 4 to 4 am the following day to accommodate social habits and intake nadirs. The degree of food processing was categorized using the NOVA classification, ranging from unprocessed or minimally processed (NOVA1) to ultra-processed (NOVA4) foods and drinks (Monteiro et al., 2018). Dietary intake was evaluated using a semi-quantitative food frequency questionnaire (FFQ) validated for the French-speaking Swiss adult population (Abreu et al., 2014; Morabia et al., 1994).

#### MS-related disability variables

Disability associated with MS was assessed using the EDSS (Meyer-Moock et al., 2014). MS-related fatigue was measured with the EMIF-SEP scale across four dimensions: cognitive, physical, social, and psychological (Pittion-Vouyovitch et al., 2006). Concentrations of sNfL and sGFAP were measured with the ultrasensitive single molecule array (SIMOA) immunoassay (Neurology 2-plex B assay, Quanterix (Kuhle et al., 2016)).

### Metabolomics analysis

Blood samples were collected after an overnight fast, fractionated according to a standard protocol, and Lithium-Heparin plasma samples were aliquoted and frozen at -80 °C. These plasma samples were then sent on dry ice to Metabolon in Durham, NC, USA, where the metabolomics analysis was conducted using ultra-high performance liquid chromatography-tandem mass spectrometry (UPLC-MS/MS) (Lawton et al., 2008). Proteins were precipitated with methanol under vigorous shaking and centrifugation. Quality control was ensured by adding recovery standards, and the resulting extract was divided into five fractions: two for analysis by reverse-phase (RP) UPLC-MS/MS with positive electrospray ionization (ESI), one for RP UPLC-MS/MS with negative ESI, one for hydrophilic interaction chromatography (HILIC) UPLC-MS/MS with negative ESI, and one reserved for backup.

All UPLC-MS/MS methods utilized a Waters ACQUITY UPLC system and a Thermo Scientific Q-Exactive Orbitrap mass spectrometer operated at 35,000 mass resolution. The system alternated between MS and data-dependent MSn scans with dynamic exclusion. The scan range generally spanned 70–1000 m/z. Gradient elution was performed using C18 or HILIC columns, depending on the method, with various combinations of methanol, acetonitrile, water, formic acid, ammonium formate, and perfluoropentanoic acid, optimized for hydrophilic or hydrophobic compound detection.

Metabolites were identified by automated comparison of ion features to a proprietary reference library of over 3,300 standards, incorporating retention time, accurate mass (± 10 ppm), and MS/MS spectral data. Peak identification was further curated by expert analysts to ensure consistency and remove artifacts.

Comprehensive quality control procedures included process blanks, pooled technical replicates from study samples, and internal/recovery standards. Instrument variability was assessed using relative standard deviation of spiked standards, while overall process variability was calculated from endogenous metabolites in pooled samples. Data were normalized using run-day median scaling to correct for inter-day variation.

### Gut microbiome

DNA was extracted from fecal samples, a negative control (PBS), and a standardized positive control (MSA-2002, ATCC, Manassas, VA) using the MagNA Pure automated platform (Roche, Basel, Switzerland). Library preparation followed the 16S Metagenomic Sequencing Library Preparation protocol (Part #15,044,223 Rev. B) from Illumina (San Diego, CA) and was quality-checked using the Fragment Analyzer with the Next Generation Sequencing Fragment Kit (AATI, Ankeny, IA). Each library preparation included at least one no-template control. All samples were normalized to 4 nM before pooling. The pooled libraries were then diluted to 10 pM and spiked with 10% PhiX, followed by sequencing of the V3-V4 regions of the 16S rRNA gene on the Illumina MiSeq platform. Paired-end reads from the 16S rRNA amplicons were processed into Amplicon Sequence Variants (ASVs) using the zAMP bioinformatic pipeline (version 0.9.15), based on DADA2 (Callahan et al., 2016). The subsequent analyses were conducted using relative abundances of bacterial genera.

### Statistical analyses

We used R version 4.3.1 for data processing, statistical modeling, and generating figures (ggplot2 package v3.4.4). To address non-normality and heteroscedasticity in our data, we applied two transformations: logarithmic (log(x + 1)) to metabolomics and clinical datasets, and standardization (log((x + 0.01)/sd(x + 0.01))) to the relative abundance bacterial dataset. We first selected bacterial genera with relative abundances differing from 0 in at least 20% of PwMS. These transformations aimed to normalize the data and stabilize variance, facilitating more robust statistical analyses.

We used the least absolute shrinkage and selection operator (LASSO) algorithm with ten-fold cross-validation (caret package, v6.0.94) to predict MS-related parameters based on plasma metabolites, gut microbiota, and clinical data. The models were adjusted for sex, age, BMI, and the four disease-modifying therapies (DMTs) classes summarized in Table 1. The optimal regularization parameter (lambda) was selected based on ten-fold cross-validation, minimizing the root mean squared error (RMSE) of predictions. We used the default lambda range within the'caret' package (which depends on the data), and the tested lambda values and RMSE for each model are summarized in Table S1. For each set of variables, we assessed the predictive performance of the LASSO model using Pearson correlation coefficients between predicted and measured values through ten-fold cross-validation. After selecting the most important variables using different LASSO models, we calculated the Spearman correlation coefficient to assess the univariate relationships between selected variables and MS-linked parameters.

## Results

### Study participants

Among the 54 PwMS included in the FoodMS cohort, one participant dropped out and was excluded from the analysis. Seventy-nine percent of participants were female, which aligns with the higher prevalence of MS in females. The median age was 34 years (interquartile range [IQR] 29—43) and the median BMI was 23.9 kg/m^2^ (IQR 21.6—26.6). Among the 53 PwMS, 51 had relapsing–remitting MS (RRMS), one had secondary progressive MS (SPMS), and one clinically isolated syndrome (CIS). The median EDSS value was 1.5 (IQR 1.0—2.0, range 0– 10, with 10 indicating the highest disability) and the median duration since diagnosis was 22 months (IQR 12—44). Forty-five participants were following treatment with different DMTs, while 8 PwMS were not on DMT at the time of the study (Table 2).

**Table 2:** Baseline clinical characteristics and MS parameters

	Persons with Multiple Sclerosis (n = 53)	
**Clinical characteristics**		
Female, no (%)	42 (79%)	
Age, years	34 (29—43)	
**MS characteristics**		
Duration since diagnosis, months	22 (12—44)	
MS disease-modifying therapies	None	8 (15.1%)
	First-line injectable (IFN, Glatiramer)	10 (18.9%)
	Oral treatments (Cytostatic agents, Fumarate, S1P blockade, Nucleoside analog)	22 (41.6%)
	Monoclonal antibodies (anti-CD20, anti-CD52, anti-VLA4)	13 (24.6%)
MS severity parameters		
EDSS^a^ score	1.5 (1.0—2.0)	
EMIF-SEP^b^ total score	35.9 (21.4—50.0)	
sNfL concentration, pg/mL	8.0 (5.5—11.2)	
sGFAP concentration, pg/mL	66.8 (48.1—82.3)	

Data are presented as median (IQR) for continuous variables, or as no. individuals (%) for categorical variables

*IQR* Interquartile range, *EDSS* Expanded Disability Status Scale, *IFN* Interferon, *MS* Multiple sclerosis, *S1P* Sphingosine-1-phosphate, *CD* Cluster of differentiation, *VLA* Very late antigen, *sNfL* Serum Neurofilament light chain, *sGFAP* Serum glial fibrillary acidic protein, *EMIF-SEP* Échelle de mesure de l’impact de la fatigue dans la sclérose en plaques; Scale for measuring the impact of fatigue in multiple sclerosis

^a^EDSS is a scale of disability in MS, ranging from 0 (normal neurological function) to 10 (death due to MS), with a 0.5 increment

^b^EMIF-SEP is an MS-related fatigue score ranging from 0 (no fatigue) to 120 (maximum fatigue)

### Metabolomics and clinical data-based models predict key parameters of MS

**Fig. 1:**
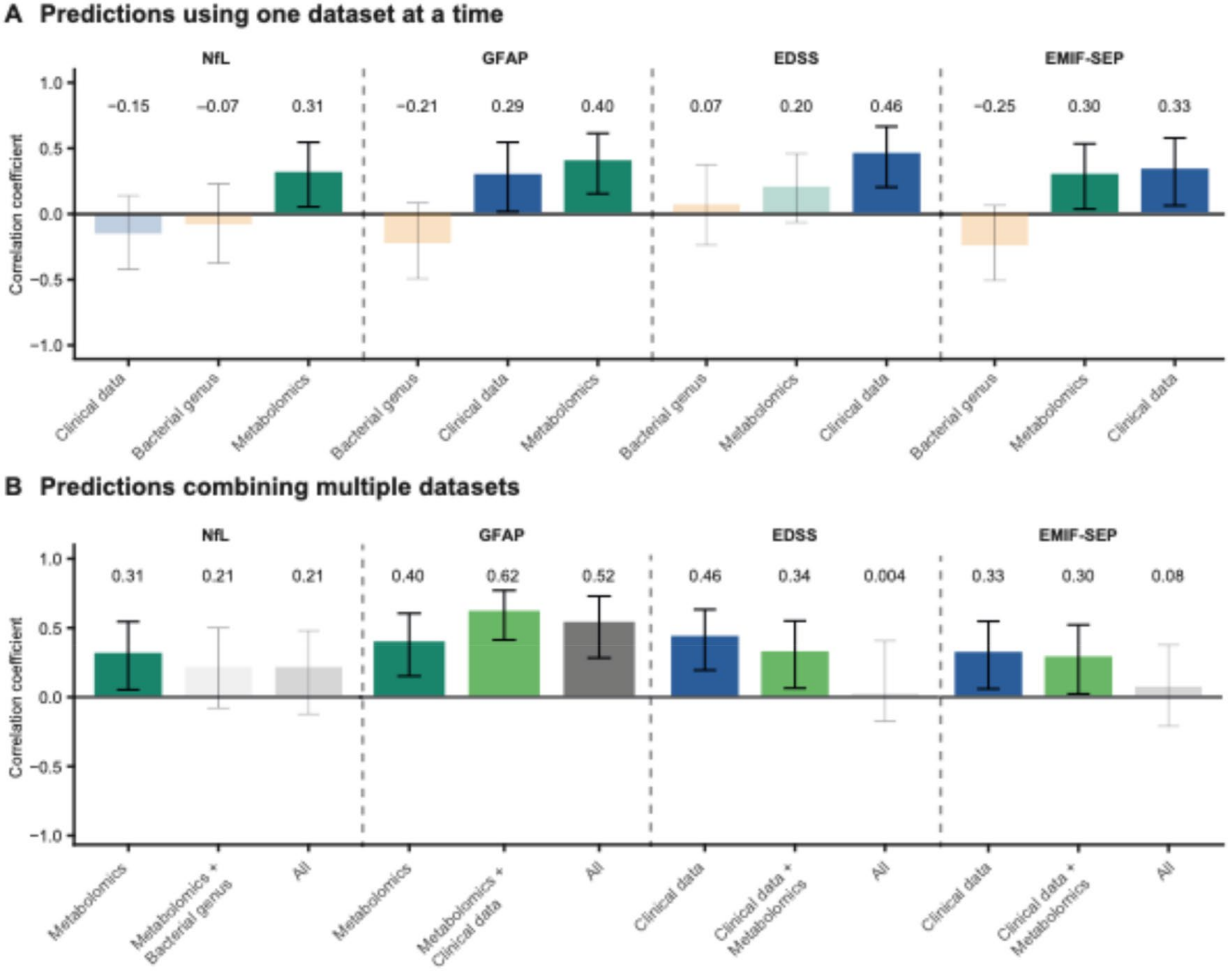
Metabolomics and clinical data are the best predictors for MS-related parameters. **A-B** Evaluation of model performance using Pearson correlation for (**A**) each model individually, or (**B**) by combining the different datasets. Non-significant models are displayed with transparency

Plasma metabolites were found to be superior predictors for sNfL and sGFAP, while clinical data showed a stronger association with EDSS (Fig. 1A). Both circulating metabolites and clinical data were significant predictors of MS-related fatigue as assessed with EMIF-SEP (Fig. 1A). Notably, gut microbiota did not significantly predict MS-related parameters (Fig. 1A).

We then investigated whether combining different datasets could strengthen the predictive performance of MS-related parameters. Integrating datasets did not improve predictive performance, as evaluated by Pearson’s correlation coefficients (Fig. 1B).

To assess if DMTs could affect metabolomic profiles between treated and untreated PwMS, we compared the global metabolomic profiles of PwMS receiving DMT to those who were untreated. Principal component analysis (PCA) did not reveal any visual separation between the two groups, and this was confirmed statistically using PERMANOVA (Figure S1A, PERMANOVA, p = 0.76). Specific DMT classes did not influenced the metabolome by comparing subgroups of PwMS according to DMT category (Figure S1B PERMANOVA, p = 0.10). These findings suggest that DMT use does not have a major impact on the overall metabolomic profile in our cohort. We also showed that taking antidepressants did not impact the metabolomic profile of PwMS by PCA and PERMANOVA (Figure S1C, PERMANOVA, p = 0.72).

As dietary changes could account for bias in metabolites and gut microbiome, we further included dietary variables (classified as Dietary intake and food quality in Table 1) as potential covariates in our LASSO models for both the metabolomic and microbiota datasets (Figure S2A). Incorporating dietary variables modestly improved the predictive performance of models based on metabolomics data and affected the predictive performance of models based on gut microbiome (Figure S2A). Correlation coefficients increased from 0.31 to 0.47 for sNfL, and from 0.40 to 0.51 for GFAP. In contrast, the addition of dietary variables had little to no effect on microbiota-based models. Diet is thus unlikely to be the main driver of the observed associations.

### Predictive features of metabolomics-based models

**Fig. 2:**
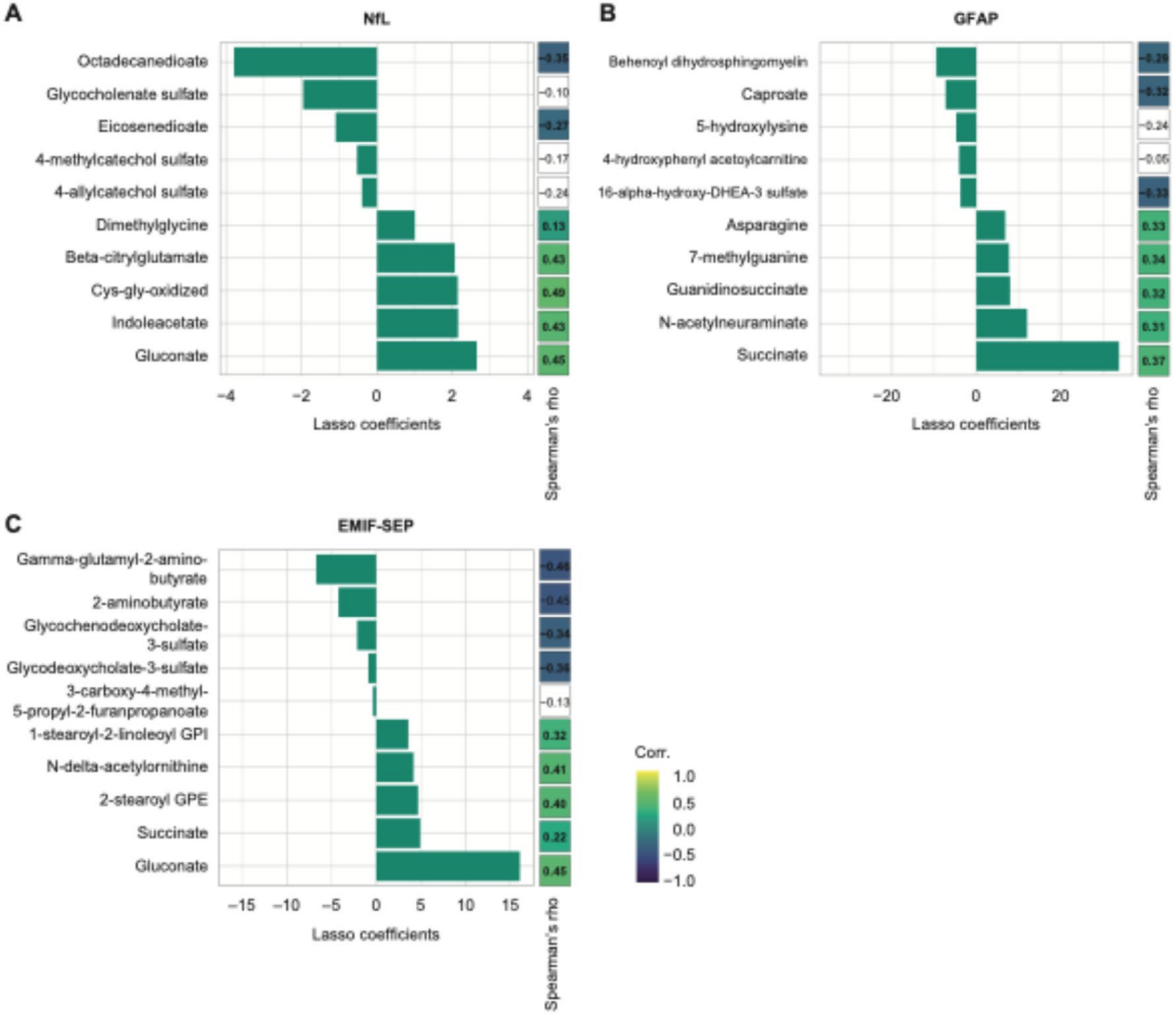
Metabolomics as a predictor of sNfL concentration, sGFAP concentration, and MS-related fatigue score. **A**-**C** Summary of the 10 most important LASSO coefficients and their univariate Spearman’s coefficient correlation for (**A**) sNfL concentration, **B** sGFAP concentration, and (**C**) MS-related fatigue (EMIF-SEP). Non-significant Spearman’s correlations are left blank

After identifying metabolomics as the most predictive dataset, we focused on its components as predictors for various MS-related parameters. Metabolomics data significantly predicted sNfL (Pearson’s r = 0.31, p = 0.02) based on 26 metabolites (Table S2). By focusing on the 10 most influential metabolites in predicting sNfL, we identified 5 metabolites with the most positive and negative LASSO coefficients (Fig. 2A). Of the 26 selected metabolites, 20 of them from diverse pathways showed a significant univariate correlation with sNfL (Fig. 2A, Table S2). Two tryptophan metabolites, indole-acetate and indole-3-carboxylate (Fig. 2A, Table S2), correlated positively with sNfL, while fatty acids eicosenedioate and octadecanedioate were negatively associated with sNfL (Fig. 2A).

Blood metabolites were the best predictors of sGFAP in PwMS (Pearson’s r = 0.39, p = 0.003). Twenty-eight metabolites out of 43 selected by the LASSO algorithm showed significant univariate correlations with sGFAP (Fig. 2B, Table S3). Succinate, N-acetylneuraminate, and N6-carboxymethyl lysine correlated positively with sGFAP and are part of carbohydrate metabolism (Table S3). Additionally, four gut-derived metabolites were highlighted by LASSO: Tryptophan, vanillactate, and lysine correlated positively with sGFAP whereas caproate correlated negatively (Table S3).

**Fig. 3:**
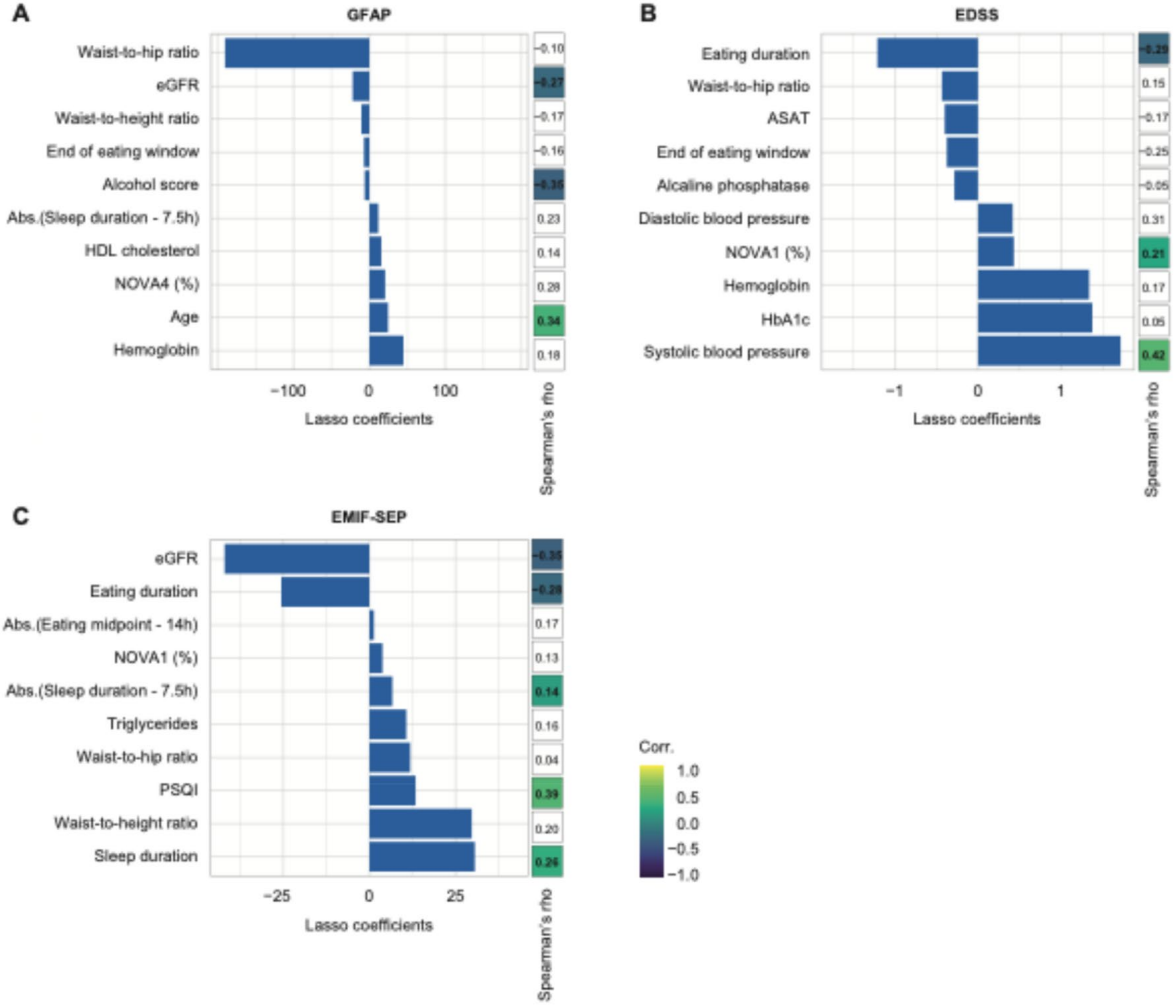
Clinical data can significantly predict sGFAP concentration, EDSS, and, MS-related fatigue score. **A**-**C** Summary of the 10 most important LASSO coefficients and their univariate Spearman’s coefficient correlation for (**A**) sGFAP concentration, **B** EDSS, and **C** MS-related fatigue (EMIF-SEP). Non-significant Spearman’s correlations are left blank. “Abs.” denotes the absolute value function

Metabolomics significantly predicted MS-related fatigue in PwMS (Pearson’s r = 0.29, p = 0.03), with 70% of metabolites identified by the LASSO algorithm also showing univariate correlations with EMIF-SEP. Interestingly, the bile acids glycochenodeoxycholate sulfate and glycodeoxycholate-3-sulfate were negatively associated with MS-related fatigue (Fig. 3C, Table S4). Furthermore, glycosyl-N-palmitoyl-sphingosine and palmitoyl-sphingosine-phosphoethanolamine were two ceramides that correlated positively with MS-related fatigue (Fig. 3C, Table S4).

### Features of the clinical-based model that predict MS biomarkers

We then addressed clinical variables including MS related disability, clinical and metabolic health, and lifestyle and dietary variables (Table 1). Clinical data significantly predicted sGFAP in PwMS (Pearson’s r = 0.29, p = 0.04). However, only 4 out of the 19 clinical variables selected by the LASSO algorithm significantly correlated with sGFAP (Fig. 3A, Table S5). Indeed, the alcohol score and eGFR correlated negatively with sGFAP (Spearman’s rho = -0.35 and − 0.27, p = 0.03 and 0.01, respectively), whereas age and sNfL correlated positively (Spearman’s rho = 0.34 and 0.32, p = 0.02 and 0.02, respectively) (Fig. 3).

Clinical variables predicted EDSS in PwMS with the highest accuracy (Fig. 3B, Pearson’s r = 0.45, p = 0.001). Out of 16 variables selected by the LASSO algorithm, 7 correlated significantly with EDSS (Fig. 3B, Table S6). Eating duration, eGFR, and IPAQ total correlated negatively with EDSS (Spearman’s rho = − 0.29; − 0.21; − 0.37, p = 0.02; 0.04; 0.02, respectively) whereas systolic blood pressure, unprocessed or minimally processed foods (NOVA1 proportion in %), mean sleep duration (absolute value of sleep duration—7.5 h) and DMT were positively associated (Spearman’s rho = 0.21 to 0.42, p = 0.02 to 0.03).

Clinical variables were good predictors of MS-related fatigue (Pearson’s r = 0.33, p = 0.02). Six out of 11 clinical variables selected by the LASSO algorithm correlated significantly with the EMIF-SEP (Fig. 3C, Table S7). Sleep duration, PSQI score, sex and mean sleep duration correlated positively with EMIF-SEP (Spearman’s rho = 0.14 to 0.38; p = 0.005 to 0.03) whereas eating duration and eGFR were negatively associated (Spearman’s rho = -0.27 and -0.35, p = 0.006 and 0.001).

### Shared predictive features of multiple MS variables

**Fig. 4:**
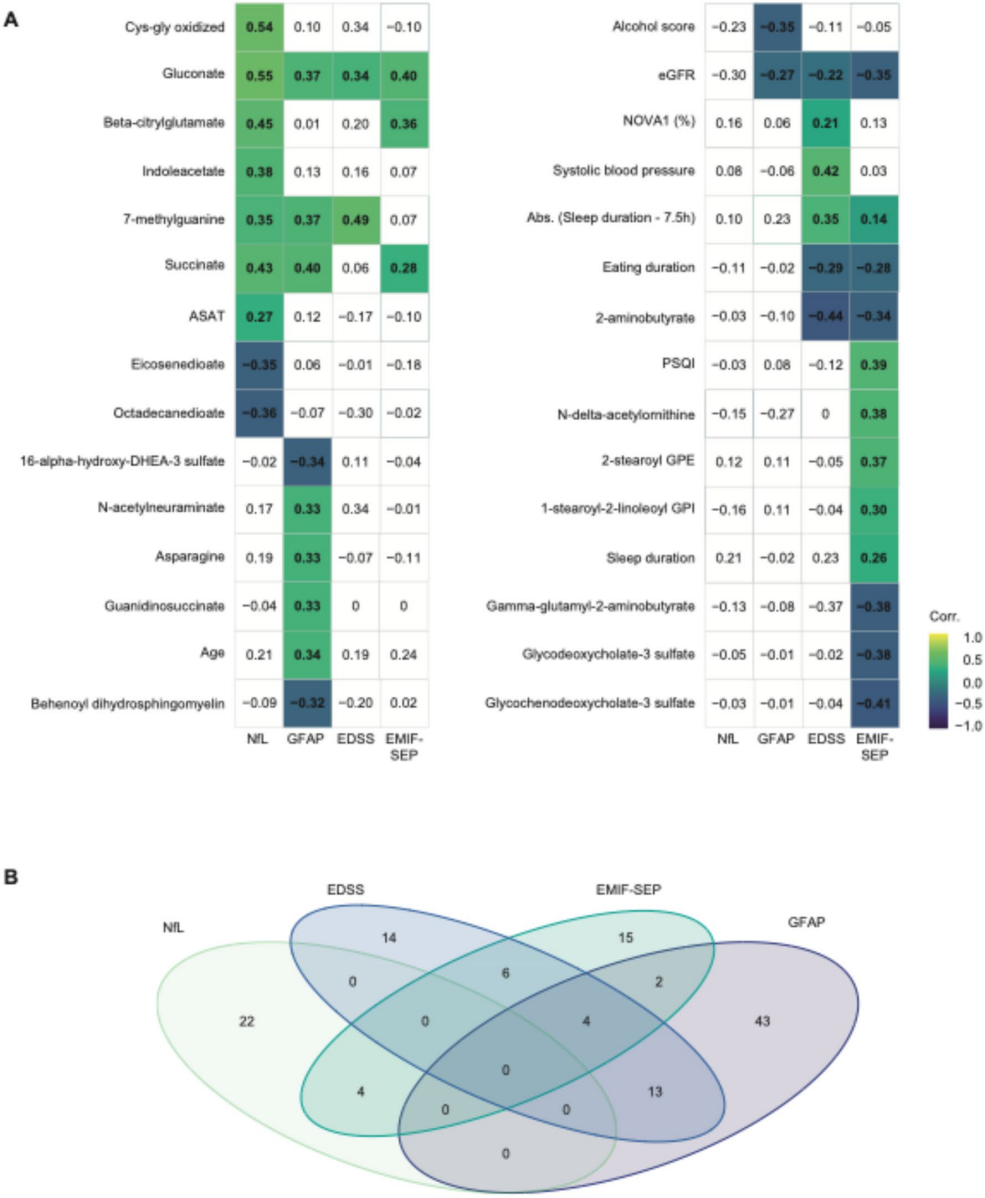
Most variables highlighted by the LASSO models are specific to one MS-related parameter. **A** Spearman’s correlation matrix of the variables selected by at least one of the LASSO models. **B** Venn Diagram showing variables shared among the four different MS-linked variables studied. Non-significant Spearman’s correlations are left blank

Finally, we identified and summarized 30 variables that were significantly correlated with at least one MS-linked parameter and selected by the LASSO algorithm in at least one of the significant models (Fig. 4A). Of the 30 variables, 20 were metabolites, highlighting the importance of metabolomics in predicting the MS-linked parameters under investigation. Visualized in a Venn diagram (Fig. 4B), we found that most selected variables were unique to a specific MS-linked parameter, with none shared across all four. However, there were some overlaps between 2 or 3 different parameters.

For example, sNfL and MS-related fatigue shared 4 metabolites: 4-methycatechol sulfate, beta-citrylglutamate, gluconate, and glycosyl-N-palmitoyl sphingosine. MS-related fatigue and sGFAP had 6 variables in common (succinate and WtHR) 4 of which were also shared with EDSS (absolute value of Sleep duration—7.5 h, eGFR, NOVA1, and WHR). Additionally, EDSS and sGFAP shared 13 variables (Fig. 4B, EMIF-SEP physical and psychological, age, alcohol score, ASAT, eating stop, fruit score, hemoglobin, sNfL, NOVA4, nut/soy score, alkaline phosphatase, and vegetable score).

While sNfL and sGFAP concentrations are both markers of brain injury, no variables were shared between the two models (Fig. 4B). However, gluconate, although not retained in sGFAP’s LASSO model, showed a positive correlation with the 4 MS-related parameters.

## Discussion

In this study, we assessed the predictive potential of metabolomics, gut microbiota, and clinical factors on MS-related fatigue, EDSS, concentrations of sNfL, and sGFAP to identify markers associated with MS disease severity. We used the LASSO algorithm to predict disease activity measurements in MS, revealing that plasma metabolites hold the most predictive potential for sNfL and sGFAP concentrations and MS-related fatigue. Our analyses were adjusted for age, BMI, and DMTs as it can impact sNfL and sGFAP concentrations (Benkert et al., 2022). We also performed a subsequent analysis assessing the impact of DMTs and dietary factors on metabolomics and gut microbiome.

Our study of recently diagnosed and mildly impaired PwMS allows for the identification of early metabolic changes. This contrasts with many studies including PwMS with longer disease duration and higher disability scores. Additionally, our approach aimed to link metabolomics with the novel biomarkers sNfL and sGFAP, as well as MS-related fatigue, whereas several studies aimed at identifying a metabolomics signature differentiating PwMS from controls, classifying PwMS according to EDSS (Villoslada et al., 2017) or MS-type (Shi et al., 2023). Furthermore, physical exercise could improve fatigue in PwMS through metabolic changes (Keller et al., 2021). These findings strengthen our results, demonstrating the critical role of metabolomics in predicting MS-related fatigue scores.

While gut microbiota species displayed no predictive capacity for any of the assessed MS-related disease parameters, several gut-derived metabolites were significantly impacted when measured in plasma. This disparity may arise from the redundancy in microbiota analyses, where overlapping bacterial functions weaken specific associations. In contrast, metabolomics directly reflects microbial and host metabolic outputs, offering a clearer and more quantifiable measure of activity. Similarly, in another study, the blood metabolome outperformed the gut microbiome in classifying PwMS and controls (Cantoni et al., 2022).

Gut-derived tryptophan metabolites, including indole-acetate and indole-3-carboxylate, showed positive correlations with sNfL concentration. Interestingly, several gut bacteria metabolize tryptophan into indole derivatives, which can have immunomodulatory functions through their interaction with the aryl hydrocarbon receptor (AhR), a transcription factor involved in CD4^+^ T-cell differentiation (Apetoh et al., 2010). Additionally, indole-acetate is positively associated with disease duration (Herman et al., 2019). Various gut-derived metabolites such as tryptophan, vanillactate, lysine, and the medium-chain fatty acid caproate correlated with sGFAP concentration. Tryptophan is an essential amino acid obtained solely from dietary sources. It can be used for protein synthesis, metabolized through the kynurenine pathway, or by gut bacteria. Tryptophan metabolism is disrupted in PwMS (Rebeaud et al., 2022). The positive correlation of tryptophan with sGFAP concentration in PwMS suggests a negative effect of tryptophan metabolism in MS. Additionally, we found that several bile acid metabolites, glycochenodeoxycholate sulfate, and glycodeoxycholate-3-sulfate, were negatively associated with MS-related fatigue, indicating a potential beneficial impact on MS-related fatigue in our cohort of PwMS. Higher levels of circulating bile acids correlated with less deterioration in clinical disability in a previous study (Cortese et al., 2022).

Moreover, several metabolites were identified as significant predictors of MS-related parameters. For example, gluconate was positively associated with all MS-related parameters, while succinate correlated positively with sNfL, sGFAP, and MS-related fatigue, and 7-methylguanine was linked with sNfL, sGFAP, and EDSS. These metabolites could serve as biomarkers of disease activity. However, their role in MS remains a subject of debate. Succinate is produced by both the host and the gut microbiota and can also be found in the diet as a food additive. It is part of the tricarboxylic acid cycle (TCA) and is a signaling molecule acting as a pro-inflammatory stimulus to regulate immune response, local stress, and tissue damage (Tannahill et al., 2013). High circulating levels of succinate have been reported in several metabolic and inflammatory-related diseases and obesity (Serena et al., 2018). Interestingly, *Akkermansia muciniphila* can produce succinate as a by-product of mucin degradation (Chia et al., 2018), and its levels are increased in PwMS (Berer et al., 2017). *Akkermansia muciniphila* induces pro-inflammatory responses in monocolonized mice and human mononuclear cells (Cekanaviciute et al., 2017). Oral supplementation of succinate enhances EAE disease severity by increasing IL-1β production and Th17 differentiation (Sugiyama et al., 2023).

Overall, our findings indicate that circulating metabolites outperformed clinical data and gut microbiota in predicting sNfL and sGFAP concentrations as well as MS-related fatigue. Although gut microbiota sequencing did not enhance the predictive power in this study, it remains a promising area for future exploration. Moreover, integrating metabolomics with gut microbiota sequencing could provide a more comprehensive understanding of the interactions between microbiota and host in MS. Blood metabolites proved to be effective and significant in the LASSO algorithm and univariate Spearman’s correlation. While clinical variables can predict sGFAP, EDSS, and MS-related fatigue, they lack strong correlations in univariate analysis. Metabolites such as gluconate, succinate, and 7-methylguanine emerged as positively associated with multiple MS-related parameters and could potentially serve as biomarkers for MS severity. Further studies are necessary to assess their predictive value in larger cohorts and to understand their underlying pathophysiological mechanisms.

While the findings of this study provide new insights, several limitations should be considered when interpreting the results. The sample size was relatively small and recruited from a single center, which may affect statistical power and limit the generalizability of the findings. The cohort was largely composed of individuals with RRMS, with minimal representation of other subtypes, which restrains the applicability of the findings to the broader MS population. The dataset was exploratory and not designed with longitudinal or interventional components, restricting the ability to draw causal inferences. The study relied on specific omics platforms and analytical standards for both metabolomic and microbiota profiling, which, although robust, may not fully capture biological variability or be directly comparable across studies. Lastly, metabolomic analyses were performed on plasma, while NfL and GFAP were measured in serum; although previous studies report high concordance between these matrices (Youssef et al., 2023), minor matrix effects cannot be entirely excluded. These limitations highlight the need for further validation in larger, more diverse cohorts with longitudinal designs.

## Supplementary Information

Below is the link to the electronic supplementary material.

Supplementary file1 (DOCX 49 KB)

Supplementary file2 (PDF 100 KB)

## Data Availability

The datasets generated during and/or analyzed during the current study will be accessible via public data repositories.
