# Supplementary material for: Blood metabolomics improves prediction of central nervous system damage in multiple sclerosis
Source: Metabolomics. 2025 Aug 12;21(5):114. doi: 10.1007/s11306-025-02315-2 (PMC12343719; doi:10.1007/s11306-025-02315-2)
Supplement: Supplementary file 1 — Supplementary file1 (DOCX 49 KB) [file 11306_2025_2315_MOESM1_ESM.docx]

**Table S1. Models’ performance summary table**.

| Model | Range of lambda values | Optimal lambda | RMSE | Pearson’s r | P-value |
| --- | --- | --- | --- | --- | --- |
| NfL - metabolomics | 0,02-2,54 | 0,50 | 4,28 | 0,31 | 0,02 |
| NfL - clinical data | 0,01-1,57 | 0,99 | 4,18 | -0,15 | 0,32 |
| NfL - bacterial genus | 0,02-2,00 | 1,26 | 4,57 | -0,07 | 0,65 |
| GFAP - metabolomics | 0,27-21,58 | 2,37 | 19,39 | 0,40 | 0,003 |
| GFAP - clinical data | 0,09-9,81 | 1,96 | 25,00 | 0,29 | 0,04 |
| GFAP - bacterial genus | 0,11-10,97 | 6,94 | 29,17 | -0,21 | 0,18 |
| EDSS - metabolomics | 0,004-0,37 | 0,23 | 0,65 | 0,20 | 0,14 |
| EDSS - clinical data | 0,02-2,88 | 0,18 | 0,74 | 0,46 | 0,001 |
| EDSS - bacterial genus | 0,03-3,03 | 0,19 | 0,77 | 0,07 | 0,62 |
| EMIF-SEP - metabolomics | 0,20-19,26 | 6,69 | 19,31 | 0,30 | 0,03 |
| EMIF-SEP - clinical data | 0,10-10,43 | 2,08 | 19,18 | 0,33 | 0,02 |
| EMIF-SEP - bacterial genus | 0,08-8,84 | 5,59 | 21,39 | -0,25 | 0,10 |
| NfL - metabolomics + bacterial genus | 0,05-4,51 | 1,56 | 4,42 | 0,21 | 0,17 |
| NfL - all | 0,06-4,48 | 1,56 | 4,43 | 0,21 | 0,17 |
| GFAP - metabolomics + clinical data | 0,34-23,95 | 0,83 | 18,45 | 0,62 | 0,00 |
| GFAP - all | 0,45-26,09 | 0,90 | 25,14 | 0,52 | 0.00 |
| EDSS - metabolomics + clinical data | 0,009-0,73 | 0,25 | 0,70 | 0,34 | 0,01 |
| EDSS - all | 0,006-0,39 | 0,24 | 0,73 | 0,004 | 0,97 |
| EMIF-SEP - metabolomics + clinical data | 0,25-18,96 | 6,56 | 20,33 | 0,30 | 0,04 |
| EMIF-SEP - all | 0,16-11,11 | 7,02 | 21,34 | 0,08 | 0,61 |

Abbreviations: EDSS, Expanded Disability Status Scale; EMIF-SEP, échelle de mesure de l’impact de la fatigue dans la sclérose en plaques; RMSE, Root mean squared error; sGFAP, serum glial fibrillary acidic protein; sNfL, serum Neurofilament light chain.

**Table S2. Variables contributing to the significative LASSO model including metabolomics data predicting sNfL concentration**

| Metabolite | Lasso coefficient | Spearman’s correlation coefficient | P-value | Super Pathway/ Class of bacteria | Sub Pathway |
| --- | --- | --- | --- | --- | --- |
| Gluconate | 2,651 | 0,452 | 0,001 | Xenobiotics | Food Component/Plant |
| Indoleacetate | 2,155 | 0,425 | 0,001 | Amino Acid | Tryptophan Metabolism |
| Cys-gly oxidized | 2,145 | 0,493 | 0,001 | Amino Acid | Glutathione Metabolism |
| Beta-citrylglutamate | 2,067 | 0,425 | 0,005 | Amino Acid | Glutamate Metabolism |
| Dimethylglycine | 1,006 | 0,128 | 0,034 | Amino Acid | Glycine, Serine and Threonine Metabolism |
| Cytidine | 0,821 | 0,287 | 0,004 | Nucleotide | Pyrimidine Metabolism, Cytidine containing |
| Glycosyl-N-palmitoylsphingosine (d18:1/16:0) | 0,741 | 0,161 | 0,406 | Lipid | Hexosylceramides (HCER) |
| Ethylmalonate | 0,698 | 0,390 | 0,049 | Amino Acid | Leucine, Isoleucine and Valine Metabolism |
| Indole-3-carboxylate | 0,662 | 0,581 | 0,001 | Amino Acid | Tryptophan Metabolism |
| N-acetylvaline | 0,473 | 0,384 | 0,001 | Amino Acid | Leucine, Isoleucine and Valine Metabolism |
| Methyl-4-hydroxybenzoate sulfate | 0,308 | 0,170 | 0,045 | Xenobiotics | Benzoate Metabolism |
| 1-3-7-trimethylurate | 0,183 | 0,199 | 0,031 | Xenobiotics | Xanthine Metabolism |
| Umbelliferone sulfate | 0,114 | 0,306 | 0,034 | Xenobiotics | Food Component/Plant |
| N6-methyladenosine | 0,085 | 0,367 | 0,028 | Nucleotide | Purine Metabolism, Adenine containing |
| Ribitol | 0,073 | 0,260 | 0,039 | Carbohydrate | Pentose Metabolism |
| Docosahexaenoylcarnitine | 0,042 | 0,187 | 0,250 | Lipid | Fatty Acid Metabolism (Acyl Carnitine, Polyunsaturated) |
| Orotate | 0,006 | 0,269 | 0,048 | Nucleotide | Pyrimidine Metabolism, Orotate containing |
| 1-2-3-benzenetriol sulfate | -0,005 | -0,170 | 0,119 | Xenobiotics | Chemical |
| 2-O-methyluridine | -0,058 | -0,068 | 0,121 | Nucleotide | Pyrimidine Metabolism, Uracil containing |
| Glucuronide of C10H18O2-7 | -0,116 | -0,146 | 0,096 | Partially Characterized Molecules | Partially Characterized Molecules |
| Glycine | -0,388 | -0,175 | 0,034 | Amino Acid | Glycine, Serine and Threonine Metabolism |
| 4-allylcatechol sulfate | -0,396 | -0,240 | 0,051 | Xenobiotics | Benzoate Metabolism |
| 4-methylcatechol sulfate | -0,530 | -0,167 | 0,138 | Xenobiotics | Benzoate Metabolism |
| Eicosenedioate | -1,096 | -0,275 | 0,032 | Lipid | Fatty Acid, Dicarboxylate |
| Glycocholenate sulfate | -1,958 | -0,100 | 0,061 | Lipid | Secondary Bile Acid Metabolism |
| Octadecanedioate | -3,797 | -0,350 | 0,003 | Lipid | Fatty Acid, Dicarboxylate |

**Table S3. Variables contributing to the significative LASSO model including metabolomics data predicting GFAP concentration**

| Metabolite | Lasso coefficient | Spearman’s correlation coefficient | P-value | Super Pathway | Sub Pathway |
| --- | --- | --- | --- | --- | --- |
| Succinate | 33,806 | 0,374 | 0,001 | Energy | TCA Cycle |
| N-acetylneuraminate | 12,030 | 0,306 | 0,014 | Carbohydrate | Aminosugar Metabolism |
| Guanidinosuccinate | 8,012 | 0,321 | 0,005 | Amino Acid | Guanidino and Acetamido Metabolism |
| 7-methylguanine | 7,734 | 0,344 | 0,006 | Nucleotide | Purine Metabolism, Guanine containing |
| Asparagine | 6,861 | 0,325 | 0,030 | Amino Acid | Alanine and Aspartate Metabolism |
| N-acetyl-2-aminoadipate | 6,385 | 0,320 | 0,072 | Amino Acid | Lysine Metabolism |
| Vanilloylglycine | 6,353 | 0,453 | 0,001 | Xenobiotics | Food Component/Plant |
| Citrulline | 5,436 | 0,253 | 0,030 | Amino Acid | Urea cycle; Arginine and Proline Metabolism |
| 1-linoleoyl-GPI (18:2) | 5,359 | 0,153 | 0,268 | Lipid | Lysophospholipid |
| Lysine | 5,041 | 0,355 | 0,023 | Amino Acid | Lysine Metabolism |
| Arginine | 3,505 | 0,446 | 0,000 | Amino Acid | Urea cycle; Arginine and Proline Metabolism |
| N-lactoyl-valine | 3,289 | 0,182 | 0,109 | Amino Acid | Lactoyl Amino Acid |
| Succinoyltaurine | 3,110 | 0,131 | 0,254 | Amino Acid | Methionine, Cysteine, SAM and Taurine Metabolism |
| 2-hydroxynervonate | 2,788 | 0,098 | 0,229 | Lipid | Fatty Acid, Monohydroxy |
| Vanillactate | 1,503 | 0,228 | 0,025 | Amino Acid | Tyrosine Metabolism |
| Saccharin | 1,451 | 0,225 | 0,046 | Xenobiotics | Food Component/Plant |
| 4-hydroxyphenylpyruvate | 1,297 | 0,222 | 0,124 | Amino Acid | Tyrosine Metabolism |
| Tryptophan | 1,076 | 0,252 | 0,041 | Amino Acid | Tryptophan Metabolism |
| 4-ethylphenylsulfate | 1,015 | 0,442 | 0,002 | Xenobiotics | Benzoate Metabolism |
| Cystathionine | 0,572 | 0,364 | 0,009 | Amino Acid | Methionine, Cysteine, SAM and Taurine Metabolism |
| Lactosyl-N.behenoyl-sphingosine (d18:1/22:0) | 0,426 | 0,221 | 0,071 | Lipid | Lactosylceramides (LCER) |
| N6-carboxymethyllysine | 0,279 | 0,360 | 0,005 | Carbohydrate | Advanced Glycation End-product |
| 4-acetylphenol sulfate | 0,089 | 0,392 | 0,077 | Xenobiotics | Benzoate Metabolism |
| 5alpha-androstan-3beta-17alpha-diol disulfate | -0,218 | -0,218 | 0,099 | Lipid | Androgenic Steroids |
| Sulfate of piperine metabolite (C16H19NO3). | -0,359 | -0,290 | 0,004 | Xenobiotics | Food Component/Plant |
| Alpha-ketobutyrate | -0,418 | -0,363 | 0,009 | Amino Acid | Methionine, Cysteine, SAM and Taurine Metabolism |
| 3-hydroxyoleoylcarnitine | -0,425 | -0,314 | 0,018 | Lipid | Fatty Acid Metabolism (Acyl Carnitine, Hydroxy) |
| Sphingomyelin (d18:0/18:0/d19:0/17:0). | -0,556 | -0,300 | 0,023 | Lipid | Dihydrosphingomyelins |
| 21-hydroxypregnenolone disulfate | -0,597 | -0,305 | 0,041 | Lipid | Pregnenolone Steroids |
| Dihomo-linolenoylcarnitine | -0,641 | -0,270 | 0,074 | Lipid | Fatty Acid Metabolism (Acyl Carnitine, Polyunsaturated) |
| N-acetyl-beta-alanine | -1,047 | -0,158 | 0,152 | Nucleotide | Pyrimidine Metabolism, Uracil containing |
| Androstenediol-3beta-17beta disulfate | -1,389 | -0,283 | 0,024 | Lipid | Androgenic Steroids |
| Piperine | -1,567 | -0,309 | 0,001 | Xenobiotics | Food Component/Plant |
| Eicosenoylcarnitine | -1,895 | -0,333 | 0,006 | Lipid | Fatty Acid Metabolism (Acyl Carnitine, Monounsaturated) |
| N-acetyltaurine | -2,180 | -0,174 | 0,203 | Amino Acid | Methionine, Cysteine, SAM and Taurine Metabolism |
| 5alpha-pregnan-diol disulfate | -2,261 | -0,374 | 0,002 | Lipid | Progestin Steroids |
| Homostachydrine | -2,804 | -0,142 | 0,198 | Xenobiotics | Food Component/Plant |
| 17alpha-hydroxypregnanolone glucuronide | -3,332 | -0,351 | 0,007 | Lipid | Pregnenolone Steroids |
| 16alpha-hydroxy-DHEA-3 sulfate | -3,772 | -0,335 | 0,005 | Lipid | Androgenic Steroids |
| 4-hydroxyphenylacetoylcarnitine | -4,086 | -0,051 | 0,339 | Amino Acid | Tyrosine Metabolism |
| 5-hydroxylysine | -4,753 | -0,241 | 0,097 | Amino Acid | Lysine Metabolism |
| Caproate | -7,269 | -0,320 | 0,035 | Lipid | Medium Chain Fatty Acid |
| Behenoyl-dihydrosphingomyelin (d18:0/22:0) | -9,468 | -0,288 | 0,027 | Lipid | Dihydrosphingomyelins |

**Table S4. Variables contributing to the significative LASSO model including metabolomics data predicting EMIF-SEP total**

| Metabolite | Lasso coefficient | Spearman’s correlation coefficient | P-value | Super Pathway | Sub Pathway |
| --- | --- | --- | --- | --- | --- |
| Gluconate | 16,060 | 0,446 | 0,000 | Xenobiotics | Food Component/Plant |
| Succinate | 4,920 | 0,219 | 0,023 | Energy | TCA Cycle |
| 2-stearoyl GPE | 4,680 | 0,398 | 0,002 | Lipid | Lysophospholipid |
| N-delta-acetylornithine | 4,172 | 0,408 | 0,001 | Amino Acid | Urea cycle; Arginine and Proline Metabolism |
| 1-stearoyl-2.linoleoyl GPI | 3,588 | 0,322 | 0,005 | Lipid | Phosphatidylinositol (PI) |
| Beta-citrylglutamate | 2,234 | 0,343 | 0,036 | Amino Acid | Glutamate Metabolism |
| Glycosyl-N-palmitoyl-sphingosine (d18:1/16:0) | 1,389 | 0,296 | 0,046 | Lipid | Hexosylceramides (HCER) |
| N-palmitoyl-sphinganine (d18:0/16:0) | 1,366 | 0,280 | 0,101 | Lipid | Dihydroceramides |
| Palmitoyl-sphingosine-phosphoethanolamine (d18:1/16:0) | 1,296 | 0,245 | 0,030 | Lipid | Ceramide PEs |
| Palmitoyl-linoleoyl glycerol | 0,906 | 0,279 | 0,031 | Lipid | Diacylglycerol |
| 1-myristoylglycerol | 0,489 | 0,349 | 0,050 | Lipid | Monoacylglycerol |
| Pantoate | 0,476 | 0,210 | 0,059 | Cofactors and Vitamins | Pantothenate and CoA Metabolism |
| N-acetyl-aspartyl glutamate | 0,400 | 0,206 | 0,100 | Amino Acid | Glutamate Metabolism |
| 4-methylcatechol sulfate | -0,082 | -0,206 | 0,199 | Xenobiotics | Benzoate Metabolism |
| Cis-urocanate | -0,130 | -0,328 | 0,048 | Amino Acid | Histidine Metabolism |
| 3-carboxy-4-methyl-5-propyl-2-furanpropanoate | -0,378 | -0,125 | 0,073 | Lipid | Fatty Acid, Dicarboxylate |
| Glycodeoxycholate-3 sulfate | -0,886 | -0,363 | 0,017 | Lipid | Secondary Bile Acid Metabolism |
| Glycochenodeoxycholate- sulfate | -2,142 | -0,339 | 0,003 | Lipid | Primary Bile Acid Metabolism |
| 2-aminobutyrate | -4,228 | -0,452 | 0,000 | Amino Acid | Glutathione Metabolism |
| Gamma-glutamyl-2-aminobutyrate | -6,724 | -0,462 | 0,000 | Peptide | Gamma-glutamyl Amino Acid |

**Table S5. Variables contributing to the significative LASSO model including clinical data predicting sGFAP concentration**

| Metabolite | Lasso coefficient | Spearman’s correlation coefficient | P-value | Super Pathway | Sub Pathway |
| --- | --- | --- | --- | --- | --- |
| Hemoglobin | 44,736 | 0,185 | 0,151 | Clinical data | Clinical data |
| Age | 24,508 | 0,342 | 0,022 | Clinical data | Clinical data |
| NOVA 4 (%) | 20,725 | 0,281 | 0,109 | Clinical data | Lifestyle data |
| HDL cholesterol | 15,673 | 0,137 | 0,254 | Clinical data | Clinical data |
| Abs.(Sleep duration - 7.5) | 11,697 | 0,226 | 0,163 | Clinical data | Lifestyle data |
| EMIF-SEP psychological | 7,107 | 0,127 | 0,070 | Clinical data | MS-linked |
| Vegetable score | 5,383 | 0,221 | 0,116 | Clinical data | Lifestyle data |
| NfL concentration | 3,719 | 0,325 | 0,025 | Clinical data | MS-linked |
| Nutsoy score | 3,567 | 0,218 | 0,088 | Clinical data | Lifestyle data |
| ASAT | 1,942 | 0,122 | 0,513 | Clinical data | Clinical data |
| NOVA 1 (%) | 1,686 | 0,058 | 0,586 | Clinical data | Lifestyle data |
| Fruit score | -0,015 | -0,012 | 0,753 | Clinical data | Lifestyle data |
| Alcaline phosphatase | -1,325 | -0,050 | 0,634 | Clinical data | Clinical data |
| EMIF-SEP physical | -2,216 | 0,024 | 0,577 | Clinical data | MS-linked |
| Alcohol score | -6,276 | -0,349 | 0,031 | Clinical data | Lifestyle data |
| Eating stop | -7,235 | -0,158 | 0,163 | Clinical data | Lifestyle data |
| Waist-to-height ratio | -10,591 | -0,168 | 0,461 | Clinical data | Clinical data |
| eGFR | -22,070 | -0,274 | 0,011 | Clinical data | Clinical data |
| Waist-to-hip ratio | -190,046 | -0,097 | 0,227 | Clinical data | Clinical data |

**Table S6. Variables contributing to the significative LASSO model including clinical data predicting EDSS**

| Metabolite | Lasso coefficient | Spearman’s correlation coefficient | P-value | Super Pathway | Sub Pathway |
| --- | --- | --- | --- | --- | --- |
| Systolic blood pressure | 1,714 | 0,419 | 0,022 | Clinical data | Clinical data |
| Glycated hemoglobin (HbA1c) | 1,375 | 0,049 | 0,380 | Clinical data | Clinical data |
| Hemoglobin | 1,336 | 0,167 | 0,225 | Clinical data | Clinical data |
| NOVA 1 (%) | 0,431 | 0,214 | 0,023 | Clinical data | Lifestyle data |
| Diastolic blood pressure | 0,417 | 0,307 | 0,035 | Clinical data | Clinical data |
| Sleep duration | 0,383 | 0,225 | 0,076 | Clinical data | Lifestyle data |
| Disease-modifying treatments | 0,284 | 0,396 | 0,005 | Clinical data | MS-linked |
| Fruit score | 0,271 | 0,302 | 0,076 | Clinical data | Lifestyle data |
| Abs.(Sleep duration - 7.5) | 0,216 | 0,353 | 0,039 | Clinical data | Lifestyle data |
| Triglycerides | 0,209 | 0,180 | 0,225 | Clinical data | Clinical data |
| Sex | 0,182 | -0,059 | 0,689 | Clinical data | Clinical data |
| Age | 0,181 | 0,188 | 0,088 | Clinical data | Clinical data |
| Leucocytes count | 0,163 | 0,081 | 0,587 | Clinical data | Clinical data |
| Total carbohydrates | 0,131 | 0,176 | 0,235 | Clinical data | FFQ |
| NfL concentration | 0,085 | 0,285 | 0,052 | Clinical data | MS-linked |
| Cereal score | 0,036 | 0,189 | 0,201 | Clinical data | FFQ |
| Glucose | 0,033 | 0,122 | 0,413 | Clinical data | Clinical data |
| Short fatty acid intake | 0,028 | 0,030 | 0,839 | Clinical data | FFQ |
| EMIF-SEP physical | 0,026 | 0,291 | 0,046 | Clinical data | MS-linked |
| Mono-unsaturated fatty acid intake | 0,023 | 0,138 | 0,353 | Clinical data | FFQ |
| EMIF-SEP psychological | 0,009 | 0,0489 | 0,743 | Clinical data | MS-linked |
| Fat intake | 0,008 | 0,090 | 0,546 | Clinical data | FFQ |
| Nutsoy score | -0,001 | 0,061 | 0,679 | Clinical data | FFQ |
| Vegetable score | -0,012 | 0,022 | 0,879 | Clinical data | FFQ |
| Red meat per month | -0,038 | -0,090 | 0,543 | Clinical data | FFQ |
| Alcohol score | -0,054 | -0,114 | 0,444 | Clinical data | FFQ |
| IPAQ total | -0,055 | -0,375 | 0,029 | Clinical data | Lifestyle data |
| Vitamin score | -0,079 | -0,063 | 0,669 | Clinical data | FFQ |
| NOVA 4 (%) | -0,085 | -0,074 | 0,618 | Clinical data | Lifestyle data |
| PSQI | -0,194 | -0,122 | 0,413 | Clinical data | Lifestyle data |
| eGFR | -0,223 | -0,218 | 0,041 | Clinical data | Clinical data |
| Abs.(Eating midpoint - 14.00) | -0,273 | -0,213 | 0,227 | Clinical data | Lifestyle data |
| Phosphatase alcaline | -0,285 | -0,053 | 0,721 | Clinical data | Clinical data |
| Eating stop | -0,376 | -0,247 | 0,288 | Clinical data | Lifestyle data |
| ASAT | -0,401 | -0,166 | 0,264 | Clinical data | Clinical data |
| Waist-to-hip ratio | -0,434 | 0,146 | 0,324 | Clinical data | Clinical data |
| Eating duration | -1,210 | -0,295 | 0,029 | Clinical data | Lifestyle data |

**Table S7. Variables contributing to the significative LASSO model including clinical data predicting EMIF-SEP total**

| Metabolite | Lasso coefficient | Spearman’s correlation coefficient | P-value | Super Pathway | Sub Pathway |
| --- | --- | --- | --- | --- | --- |
| Sleep duration | 30,545 | 0,261 | 0,009 | Clinical data | Lifestyle data |
| Waist-to-height ratio | 29,655 | 0,196 | 0,318 | Clinical data | Clinical data |
| PSQI | 13,374 | 0,388 | 0,005 | Clinical data | Lifestyle data |
| Waist-to-hip ratio | 11,808 | 0,036 | 0,982 | Clinical data | Clinical data |
| Triglycerides | 10,709 | 0,160 | 0,199 | Clinical data | Clinical data |
| Sex | 9,793 | 0,381 | 0,009 | Clinical data | Clinical data |
| Abs.(Sleep duration - 7.5) | 6,659 | 0,144 | 0,032 | Clinical data | Lifestyle data |
| Nova 1 (%) | 3,800 | 0,128 | 0,201 | Clinical data | Lifestyle data |
| Abs. (Eating midpoint - 14.00) | 1,305 | 0,173 | 0,306 | Clinical data | Lifestyle data |
| Eating duration | -25,553 | -0,279 | 0,000 | Clinical data | Lifestyle data |
| eGFR | -41,966 | -0,351 | 0,006 | Clinical data | Clinical data |
