# Supplementary material for: Blood metabolomics improves prediction of central nervous system damage in multiple sclerosis
Source: Metabolomics. 2025 Aug 12;21(5):114. doi: 10.1007/s11306-025-02315-2 (PMC12343719; doi:10.1007/s11306-025-02315-2)
Supplement: Supplementary file 2 — Supplementary file2 (PDF 100 KB) [file 11306_2025_2315_MOESM2_ESM.pdf]

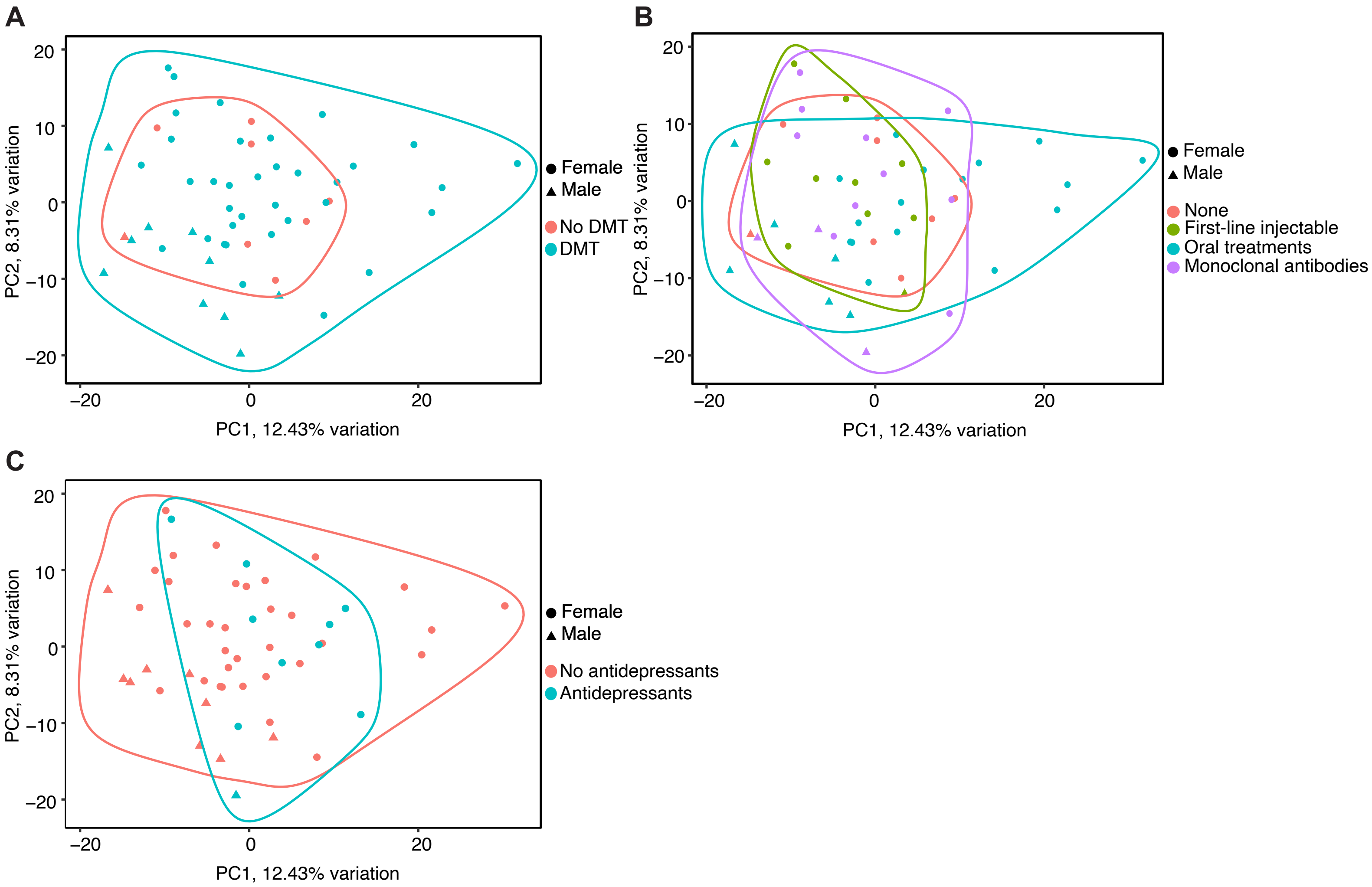

Supplementary Figure 1. Metabolomics profiles are not impacted by the different treatments. (A-B) Principal component analysis (PCA) of the plasma metabolites composition of PwMS discriminating (A) PwMS with or without treatment, (B) PwMS with the different class of DMT or (C) taking antidepressants or not.

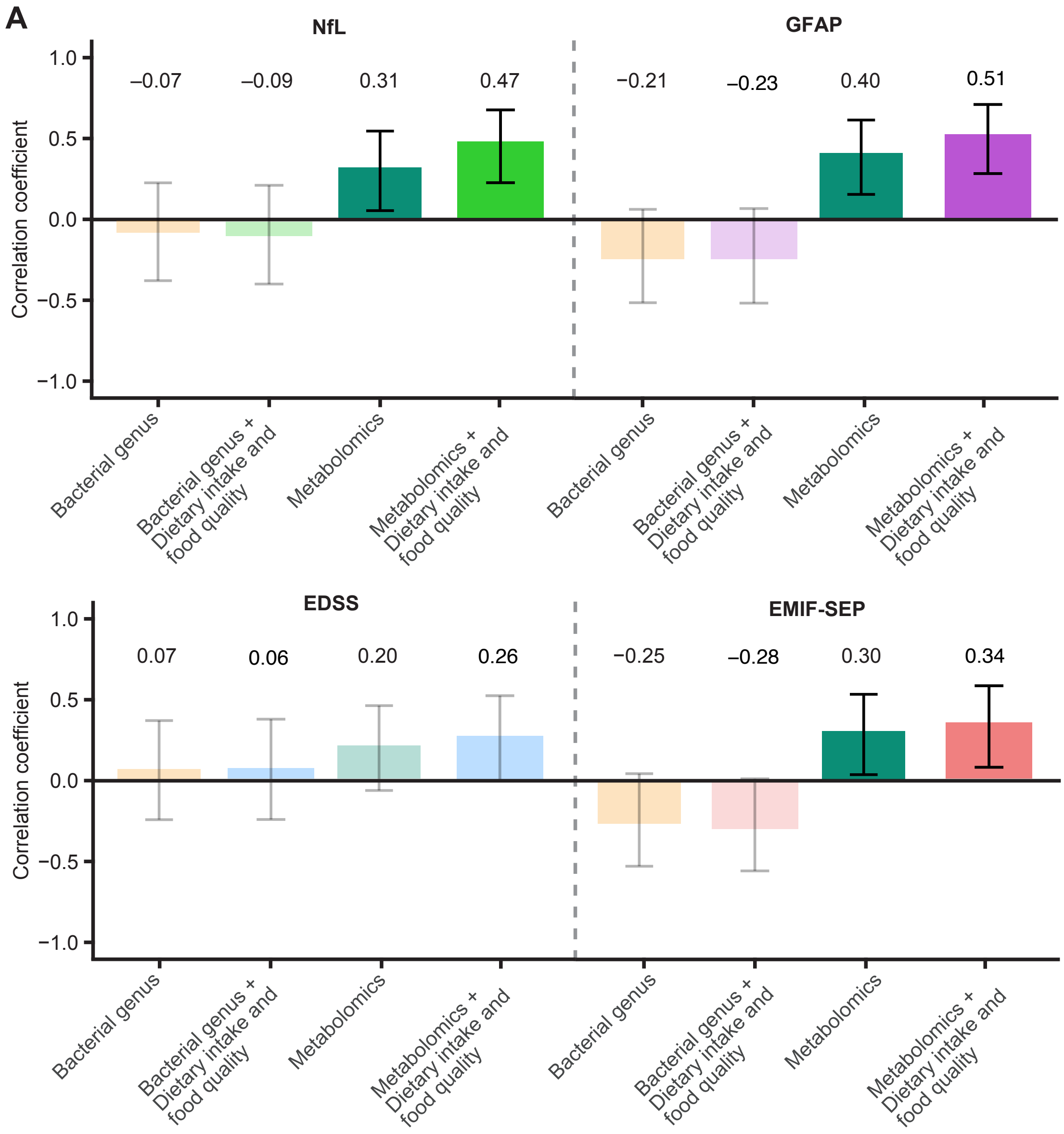

Supplementary Figure 2. Incorporating dietary intake and food quality variables does not improve the predictive performance of models based on metabolomics or gut microbiome data. (A) Model performance assessed by Pearson's correlation coefficient for each MS-related outcome, comparing models including only bacterial genera or metabolites with those incorporating dietary intake and food quality variables. Non-significant models are shown with translucent bars.
